# Complete Genome Sequence of Temperate Stenotrophomonas maltophilia Bacteriophage DLP5

**DOI:** 10.1128/genomeA.00073-18

**Published:** 2018-03-01

**Authors:** Danielle L. Peters, Jonathan J. Dennis

**Affiliations:** a Department of Biological Sciences, University of Alberta, Edmonton, Canada

## Abstract

Stenotrophomonas maltophilia bacteriophage DLP5 is a temperate phage with Siphoviridae family morphotype. DLP5 (vB_SmaS_DLP_5) is the first S. maltophilia phage shown to exist as a phagemid. The DLP5 genome is 96,542 bp, encoding 149 open reading frames (ORFs), including four tRNAs. Genomic characterization reveals moron genes potentially involved in host cell membrane modification.

## GENOME ANNOUNCEMENT

Stenotrophomonas maltophilia is an aerobic, opportunistic Gram-negative bacterium ubiquitous in aqueous environments, soils, plant rhizospheres, and hospital settings (1). S. maltophilia is capable of causing a variety of infections, and limited treatment options exist due to S. maltophilia’s exquisite innate multidrug resistance to a broad array of antibiotics (1–3). As an alternative to antibiotics, phages are being examined for treatment of S. maltophilia infections, with initial focus on phage isolation and characterization (4–16).

Phage DLP5 (vB_SmaS_DLP_5) was isolated from garden soil using S. maltophilia host strain D1614. Transmission electron micrographs identify DLP5 as a B1 morphotype Siphoviridae phage. DLP5 possesses relatively narrow tropism, infecting 5/27 clinical isolates tested. DLP5 forms clear plaques with defined boarders with an average size of 0.5 ± 0.2 mm, and one-step growth curves exhibit average burst sizes of 36. As a prophage, DLP5 replicates as a phagemid. Restriction fragment length polymorphism (RFLP) analysis suggests that the DNA is heavily modified; only 4/36 endonucleases tested could cut the DLP5 genome.

Genomic DNA was isolated from phage lysates using the Wizard DNA purification system and a modified protocol (17). A Nextera XT library was generated for paired-end sequencing on MiSeq (Illumina) platform using MiSeq v2 reagent kit and reads assembled using SPAdes 3.8.0 (18). The assembly was confirmed with PCR using 15 primer pairs randomly spaced throughout the genome with Sanger sequencing of PCR products. Open reading frames (ORFs) were identified using Glimmer (19) for Geneious (20) (Bacteria/Archaea setting) and Gene MarkS (21) for phage. The contig was annotated using BLASTP (22) and conserved domains were identified with CD-Search (23).

The phage DLP5 genome is 96,542 bp long (295-fold coverage), with GC content of 58.4%, and encoding 149 ORFs and 4 tRNAs (Sup-CTA, Glu-TTC, Gly-TCC, and Ser-GCT). Only 39 ORFs were classified with putative functions based on BLASTP analysis. DLP5 is predicted to encode two proteins involved in chromosome partitioning due to the presence of a ParBc superfamily domain; one ParBc protein also has a predicted SpoOJ superfamily domain. DNA replication, transcription, and repair proteins of interest include DNA polymerase I, DnaB, DnaG, superfamily II DNA/RNA helicase, DNA ligase, RecA, RuvC, RNase E, transcriptional regulator, transcriptional repressor, thymidylate synthase, phosphoglycerate kinase, UDP-glucose 4-epimerase, WcaG, tyrosine phosphatase, and pyruvate phosphate dikinase.

Structure/packaging predicted proteins include portal protein, large terminase subunit, major capsid protein, three tail assembly proteins, a tail fiber protein, a tape measure protein, and lysozyme. DLP5 also encodes six moron genes, which are potential virulence factors, serine protease XkdF, SAM methyltransferase, rhomboid membrane protein, PIG-L family deacetylase, WecE, and an SPFH domain-containing protein. There are also three domain-of-unknown-function proteins encoded, DUF2500, DUF3310, and DUF1643. BLASTN analysis (22) shows DLP5 is relatively unrelated to other phages, exhibiting maximum similarity of 2% with Xylella fastidiosa phage Sano (24). DLP5 genome analysis provides insight into its characteristics and potential contributions to S. maltophilia hosts during lysogeny.

### Accession number(s).

The complete genomic sequence of S. maltophilia phage vB_SmaS_DLP_5 can be accessed in GenBank under the accession number MG189906.

## References

[1] Brooke JS 2012 *Stenotrophomonas maltophilia*: an emerging global opportunistic pathogen. Clin Microbiol Rev 25:2–41. doi:10.1128/CMR.00019-11. 22232370 PMC3255966

[2] Chang YT, Lin CY, Chen YH, Hsueh PR 2015 Update on infections caused by *Stenotrophomonas maltophilia* with particular attention to resistance mechanisms and therapeutic options. Front Microbiol 6:893. doi:10.3389/fmicb.2015.00893. 26388847 PMC4557615

[3] Denton M, Kerr KG 1998 Microbiological and clinical aspects of infection associated with *Stenotrophomonas maltophilia*. Clin Microbiol Rev 11:57–80. 9457429 10.1128/cmr.11.1.57 PMC121376

[4] Semler DD, Lynch KH, Dennis JJ 2012 The promise of bacteriophage therapy for *Burkholderia cepacia* complex respiratory infections. Front Cell Infect Microbiol 1:27. doi:10.3389/fcimb.2011.00027. 22919592 PMC3417384

[5] Huang Y, Fan H, Pei G, Fan H, Zhang Z, An X, Mi Z, Shi T, Tong Y 2012 Complete genome sequence of IME15, the first T7-like bacteriophage lytic to pan-antibiotic-resistant *Stenotrophomonas maltophilia*. J Virol 86:13839–13840. doi:10.1128/JVI.02661-12. 23166248 PMC3503069

[6] Fan H, Huang Y, Mi Z, Yin X, Wang L, Fan H, Zhang Z, An X, Chen J, Tong Y 2012 Complete genome sequence of IME13, a *Stenotrophomonas maltophilia* bacteriophage with large burst size and unique plaque polymorphism. J Virol 86:11392–11393. doi:10.1128/JVI.01908-12. 22997416 PMC3457173

[7] Peters DL, Stothard P, Dennis JJ 2017 The isolation and characterization of *Stenotrophomonas maltophilia* T4-like bacteriophage DLP6. PLoS One 12:1–21. doi:10.1371/journal.pone.0173341. PMC5349666 28291834

[8] Peters DL, Lynch KH, Stothard P, Dennis JJ 2015 The isolation and characterization of two *Stenotrophomonas maltophilia* bacteriophages capable of cross-taxonomic order infectivity. BMC Genomics 16:664. doi:10.1186/s12864-015-1848-y. 26335566 PMC4559383

[9] Chen CR, Lin CH, Lin JW, Chang CI, Tseng YH, Weng SF 2007 Characterization of a novel T4-type *Stenotrophomonas maltophilia* virulent phage Smp14. Arch Microbiol 188:191–197. doi:10.1007/s00203-007-0238-5. 17440710

[10] García P, Monjardín C, Martín R, Madera C, Soberón N, Garcia E, Meana A, Suárez JE 2008 Isolation of new *Stenotrophomonas* bacteriophages and genomic characterization of temperate phage S1. Appl Environ Microbiol 74:7552–7560. doi:10.1128/AEM.01709-08. 18952876 PMC2607143

[11] Yang H, Liang L, Lin S, Jia S 2010 Isolation and characterization of a virulent bacteriophage AB1 of *Acinetobacter baumannii*. BMC Microbiol 10:131. doi:10.1186/1471-2180-10-131. 20426877 PMC2874798

[12] Chang HC, Chen CR, Lin JW, Shen GH, Chang KM, Tseng YH, Weng SF 2005 Isolation and characterization of novel giant *Stenotrophomonas maltophilia* phage φ SMA5. Appl Environ Microbiol 71:1387–1393. doi:10.1128/AEM.71.3.1387-1393.2005. 15746341 PMC1065149

[13] Hagemann M, Hasse D, Berg G 2006 Detection of a phage genome carrying a zonula occludens like toxin gene (*zot*) in clinical isolates of *Stenotrophomonas maltophilia*. Arch Microbiol 185:449–458. doi:10.1007/s00203-006-0115-7. 16775751

[14] Lee C-N, Tseng T-T, Chang H-C, Lin J-W, Weng S-F 2014 Genomic sequence of temperate phage Smp131 of *Stenotrophomonas maltophilia* that has similar prophages in *Xanthomonads*. BMC Microbiol 14:17. doi:10.1186/1471-2180-14-17. 24472137 PMC3931495

[15] Petrova M, Shcherbatova N, Kurakov A, Mindlin S 2014 Genomic characterization and integrative properties of phiSMA6 and phiSMA7, two novel filamentous bacteriophages of *Stenotrophomonas maltophilia*. Arch Virol 159:1293–1303. doi:10.1007/s00705-013-1882-5. 24327089

[16] Liu J, Liu Q, Shen P, Huang Y-P 2012 Isolation and characterization of a novel filamentous phage from *Stenotrophomonas maltophilia*. Arch Virol 157:1643–1650. doi:10.1007/s00705-012-1305-z. 22614810

[17] Lynch KH, Abdu AH, Schobert M, Dennis JJ 2013 Genomic characterization of JG068, a novel virulent podovirus active against *Burkholderia cenocepacia*. BMC Genomics 14:574. doi:10.1186/1471-2164-14-574. 23978260 PMC3765740

[18] Bankevich A, Nurk S, Antipov D, Gurevich AA, Dvorkin M, Kulikov AS, Lesin VM, Nikolenko SI, Pham S, Prjibelski AD, Pyshkin AV, Sirotkin AV, Vyahhi N, Tesler G, Alekseyev MA, Pevzner PA 2012 SPAdes: a new genome assembly algorithm and its applications to single-cell sequencing. J Comput Biol 19:455–477. doi:10.1089/cmb.2012.0021. 22506599 PMC3342519

[19] Delcher AL, Bratke KA, Powers EC, Salzberg SL 2007 Identifying bacterial genes and endosymbiont DNA with Glimmer. Bioinformatics 23:673–679. doi:10.1093/bioinformatics/btm009. 17237039 PMC2387122

[20] Kearse M, Moir R, Wilson A, Stones-Havas S, Cheung M, Sturrock S, Buxton S, Cooper A, Markowitz S, Duran C, Thierer T, Ashton B, Meintjes P, Drummond A 2012 Geneious basic: an integrated and extendable desktop software platform for the organization and analysis of sequence data. Bioinformatics 28:1647–1649. doi:10.1093/bioinformatics/bts199. 22543367 PMC3371832

[21] Besemer J, Lomsadze A, Borodovsky M 2001 GeneMarkS: a self-training method for prediction of gene starts in microbial genomes. Implications for finding sequence motifs in regulatory regions. Nucleic Acids Res 29:2607–2618. doi:10.1093/nar/29.12.2607. 11410670 PMC55746

[22] Altschul SF, Madden TL, Schäffer AA, Zhang J, Zhang Z, Miller W, Lipman DJ 1997 Gapped BLAST and PSI-BLAST: a new generation of protein database search programs. Nucleic Acids Res 25:3389–3402. doi:10.1093/nar/25.17.3389. 9254694 PMC146917

[23] Marchler-Bauer A, Lu S, Anderson JB, Chitsaz F, Derbyshire MK, DeWeese-Scott C, Fong JH, Geer LY, Geer RC, Gonzales NR, Gwadz M, Hurwitz DI, Jackson JD, Ke Z, Lanczycki CJ, Lu F, Marchler GH, Mullokandov M, Omelchenko MV, Robertson CL, Song JS, Thanki N, Yamashita RA, Zhang D, Zhang N, Zheng C, Bryant SH 2011 CDD: a conserved domain database for the functional annotation of proteins. Nucleic Acids Res 39:D225–D229. doi:10.1093/nar/gkq1189. 21109532 PMC3013737

[24] Ahern SJ, Das M, Bhowmick TS, Young R, Gonzalez CF 2014 Characterization of novel virulent broad-host-range phages of *Xylella fastidiosa* and *Xanthomonas*. J Bacteriol 196:459–471. doi:10.1128/JB.01080-13. 24214944 PMC3911242

